# Synanthropy and diversity of Phlebotominae in an area of intense transmission of visceral leishmaniasis in the South Pantanal floodplain, Midwest Brazil

**DOI:** 10.1371/journal.pone.0215741

**Published:** 2019-05-14

**Authors:** Suellem Petilim Gomes Barrios, Luciana Escalante Pereira, Neiva Zandonaide Nazário Monaco, Gustavo Graciolli, Aline Etelvina Casaril, Jucelei de Oliveira Moura Infran, Everton Falcão de Oliveira, Wagner de Souza Fernandes, Antônio Conceição Paranhos Filho, Alessandra Gutierrez de Oliveira

**Affiliations:** 1 Programa de Pós-Graduação em Doenças Infecciosas e Parasitárias, Universidade Federal de Mato Grosso do Sul, Campo Grande, Mato Grosso do Sul, Brasil; 2 Programa de Pós-Graduação em Tecnologias Ambientais, Universidade Federal de Mato Grosso do Sul, Campo Grande, Mato Grosso do Sul, Brasil; 3 Centro de Controle de Vetores, Secretaria Municipal de Saúde, Corumbá, Mato Grosso do Sul, Brasil; 4 Instituto de Biociências, Universidade Federal de Mato Grosso do Sul, Campo Grande, Mato Grosso do Sul, Brasil; 5 Instituto Integrado de Saúde, Universidade Federal de Mato Grosso do Sul, Campo Grande, Mato Grosso do Sul, Brasil; 6 Faculdade de Engenharia, Arquitetura e Urbanismo e Geografia, Universidade Federal de Mato Grosso do Sul, Campo Grande, Mato Grosso do Sul, Brasil; Instituto de Pesquisas de Rene Rachou, BRAZIL

## Abstract

Phlebotomines have been recorded from a wide variety of habitats, and some of these vector species have shown preference for human environments, with high levels of adaptation. This study evaluated the degree of preference of these vectors for urban, rural, and forested environments (synanthropic behavior), as well as the diversity of these species, in three areas (forested, rural, and urban, exhibiting different degrees of anthropogenic changes) in a region of intense transmission of visceral leishmaniasis in Corumbá county, Mato Grosso do Sul, Brazil. Using light traps, sand fly specimens were collected from the three environments simultaneously, from May 2015 to April 2017, totaling 7 213 sand flies of 14 species in eight genera. Nuorteva’s synanthropy index was determined for the species *Lutzomyia cruzi*, *Brumptomyia brumpti*, *Micropygomyia peresi*, *Lu*. *forattinii*, *Martinsmyia oliveirai* and *Evandromyia corumbaensis*. *Lutzomyia cruzi*, the vector of *Leishmania infantum* in Corumbá, was the most abundant vector species, recorded from all three areas and sampling plots, on all 24 months investigated. This species exhibited the highest synanthropic index (+75.09), indicating a strong preference for urban environments. *Brumptomyia brumpti*, *Micropygomyia peresi*, *Lu*. *forattinii*, and *Martinsmyia oliveirai* showed preference, albeit not strong, for urban environments. Overall, males were more abundant than females (*W* = 490; *p* < 0.0001). High density, high synanthropic index, and sustained indoor presence were found for *Mi*. *peresi* in the rural area. Monitoring changes in the ecological behavior of sand flies is of vital importance, as these changes may indicate an increased likelihood of leishmaniasis emergence or reemergence.

## Introduction

Leishmaniasis, a disease whose etiological agents involve roughly 20 species of the genus *Leishmania* (Kinetoplastida: Trypanosomatidae), is transmitted to animals and humans through the bites of female insects of the order Diptera, family Psychodidae, subfamily Phlebotominae. Six countries alone account for 90% of all cases of visceral and tegumentary leishmaniasis in the world. In the Americas, Brazil is the only country on this list [1, 2].

In the 1930s, incidence in Brazil was sporadic, with no epidemic cycles, but affected all age ranges. Early findings by Chagas [3] and Deane and Deane [4] led to a false belief that sand flies were restricted to wild and rural environments in the vicinity of forests. In the 1950s, however, the urbanization of leishmaniasis became evident, with cases detected in northeastern Brazil and later across the country [5–8]. Since then, epidemiological studies have revealed changes in the epidemiological profile of leishmaniasis in Brazil, formerly affecting only animals in the wild and occasionally humans who ventured into wild environments [5, 9]. The disease has now been reported from both rural and urban environments in all Brazilian regions, characterizing the entire country as an endemic area [1].

In early studies on the phlebotomine fauna of Mato Grosso do Sul state, conducted in the 1930s, sand flies were only found in the wild [3,10,11,12,13]. Unplanned urbanization, deforestation, slash-and-burn, poor sanitary conditions, and climate change have all contributed to destroying the natural habitats of sand flies, forcing these insects to seek food and shelter in human dwellings, as well as where domestic animals are housed [14].

Subsequent adaptation to these new environments has been revealed by synanthropy studies that evaluated the level of adaptation of these insects to human environments [15, 16]. Some specimens can become highly adapted to new environments, to the point of fully depending on the ecological relationships operating in the anthropogenically altered environment [16]. Varying degrees of domesticity have been observed among insect species [16].

The destruction not only of wild biocenosis, but also of shelter availability and intra- and peridomestic food sources, can foster adaptation in many insects, particularly in species exhibiting eclectic dietary behavior [17]. To measure the degree of association of a given species with human-modified environments, Nuorteva [15] developed an index that compares quantitative data for the species in three ecological areas.

The purpose of the present study was to describe the degrees of synanthropy, diversity, and richness of the phlebotomine fauna of three areas (forested, rural, and urban) along a transect exhibiting varying degrees of anthropogenic interference in Corumbá county, Mato Grosso do Sul state, Midwest Brazil, and to examine the influence of environmental variables (temperature and precipitation) on species composition.

## Methods

### Study area

**Fig 1:**
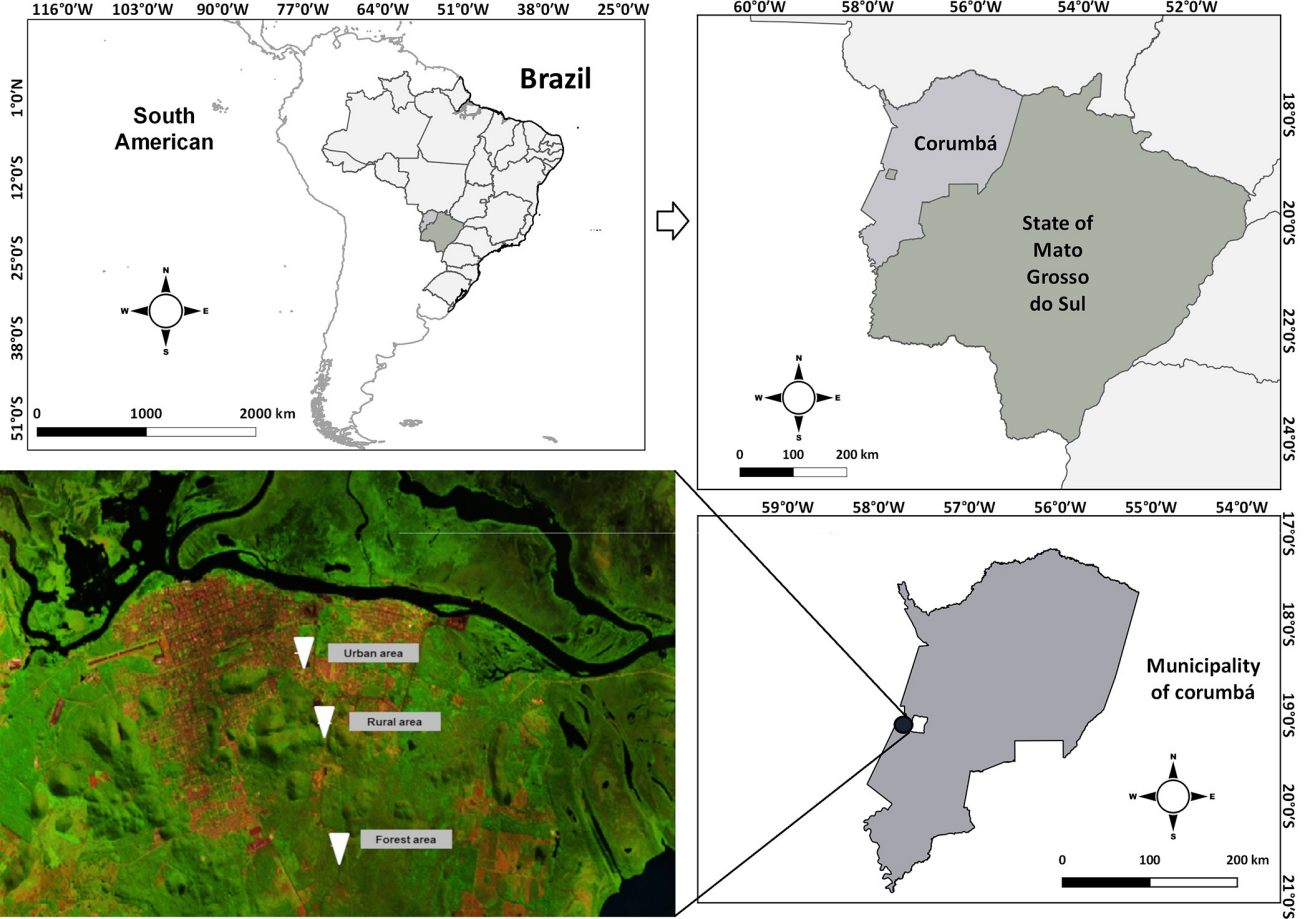
Location of the study area, with county limits and urban area of Corumbá, Mato Grosso do Sul, Brazil. White dots represent transect. The urban area of the municipality of Corumbá is represented by a Landsat 8, OLI, Band 4, 5 and 6. Earth Explorer Path 227 row 73 composition RGB 654 (26/05/2015). Note: the map of Brazil (and the shapefile used to generate it) used for the elaboration of Fig 1 was extracted from the database of public domain of the Brazilian Institute of Geography and Statistics (https://downloads.ibge.gov.br/downloads_top.php) and U.S. Geological Survey (http://viewer.nationalmap.gov/viewer/).

The municipality of Corumbá (Fig 1) (18° 59′ 44″ S; 57° 19′ 36″ W; 116 m above sea level) is located at the northwest area of Mato Grosso do Sul, Brazil, in the South-Mato-Grosso Pantanal’s plain. Corumbá is located 415 km from the State capital, Campo Grande. The city has a total area of 64,721.719 km^2^, which represents 18.19% of the State’s territorial extension. According to the Brazilian Institute of Geography and Statistics (IBGE), in 2016, the total population of Corumbá was estimated at 103,703 inhabitants and 90% of these resided in the city’s urban perimeter [18].

At the west border of Corumbá is Bolivia, which is separated from the municipality by dry border, at south-west border is Paraguay, at north is Mato Grosso State, at southeast is Porto Murtinho and at east Corumbá borders the following municipalities: Ladário, Bodoquena, Miranda, Aquidauana, Rio Verde do Mato Grosso, Coxim and Sonora. The urban area of the municipality is located in a mountainous region known as *Morraria do Urucum*, in an area of submontane deciduous forest. The predominant vegetal coverage is the Brazilian Cerrado, which is a savannah-like biome typical of the Pantanal wetland, with flat areas of low, fire-resistant trees, small palms and thorny bushes and contain characteristic vegetative formations (Cerradão, Campo Sujo, Campo Limpo and others) [19]. The Pantanal is the largest wetland in the world, of which comprise the central floodplain in Brasil, Bolivia, and Paraguay and the lowlands of the Pantanal form a broad plain. During the rainy season flooding is extensive, creating an interconnected waterway among swamps, lagoons and oxbow lakes, serve as nursery areas for fishes, crustaceans, and a variety of aquatic plants [20]. The climate is classified according to Köppen as tropical (AW), with dry winter (April to September) and rainy summer (October to March) [21, 22]. The municipality is epidemiologically classified as area of intense transmission of VL human cases [23]. From 2010 to 2017, 66 human cases of VL were confirmed in Corumbá, [24].

### Collection sites

Points were selected, along a transect, from the most preserved area (forest area), to the less preserved area (urban area) (Fig 1), totalizing three areas for sand flies collections: forest area, rural area and urban area [15] (Table 1).

**Table 1:** Coordinates and description of sampling plots (forested, rural, and urban areas). Corumbá, Mato Grosso do Sul, Brazil. May 2015 to April 2017.

Sampling plot	Coordinates	Altitude	Description	Presence of animals near insect trap
Plot 1: Forested area	19°03'14''S 57°36'54''W	233	Forest edge	Canids, birds, equines
Plot 2: Forested area	19°03'15''S 57°36'55''W	237	Forest interior, 1 m above ground	Wild birds and mammals
Plot 3: Forested area	19°03'17''S 57°36'57''W	236	Forest interior, 2 m above ground	Wild birds and mammals
Plot 4: Rural area	19°01'41''S 57°37'08''W	182	Intradomestic	Dogs, cats
Plot 5: Rural area	19°01'41''S 57°37'08''W	182	Peridomestic	Dogs, cats
Plot 6: Rural area	19°01'41''S 57°37'08''W	183	Peridomestic, forest edge	Dogs, cats
Plot 7: Urban area	19°00'45''S 57°37'30''W	154	Peridomestic, chicken coop	Dogs, birds, rabbits
Plot 8: Urban area	19°00'45''S 57°37'30''W	154	Peridomestic, open-air coop	Dogs, birds, rabbits
Plot 9: Urban area	19°00'45''S 57°37'30''W	154	Peridomestic, garden	Dogs, birds, rabbits

- Forest Area (19° 03' 14'' S; 57° 36' 54'' W): Composed of rocky areas, shrubs, trees, vines and typical herbs of *cerrado*. It is characterized as a legal reserve area, located three kilometers away from the rural area. A trap was installed at the border of the forest, intermediate site, between wild and anthropized environment (Plot 1). The other two traps were installed inside the wild forest, a meter and 2 meters above the ground (Plot 2 and 3) (Table 1).
- Rural Area (19° 01' 41' 'S; 57° 37' 08'' W): Area with a residence located near the forest area, which presents remnants of native vegetation. Presence of a dog inside the residence. The distance between rural and urban areas was 2 km. A trap was installed in the intradomicile, with presence of dogs and cats in the interior (Plot 4). Trap 5 (Plot 5) was installed in the peridomicile, with dogs and cats present, and trap 6 (Plot 6) was installed in the peridomiciliary area, on the edge of a forest adjacent to a rural residence (Table 1).
- Urban Area (19° 00' 45'' S; 57° 37' 30'' W): Area within the urban perimeter of Corumbá, with the presence of domestic animals (chickens, geese, guinea fowl, peacocks, birds and dogs) and urban lighting interference. All traps were installed in the peridomiciliary area. Trap 7 (Plot 7) was installed inside a chicken coop (*Gallus gallus domesticus*) (Linnaeus, 1758). Trap 8 (Plot 8) was attached to the birding area, such as turkey (*Meleagris* sp.) (Linnaeus, 1758) and peacock (*Pavo* sp.) (Linnaeus, 1758) and trap 9 (Plot 9) was installed in the backyard of the residence, with dogs, rabbits and the presence of leaves and fruits in decomposition on the ground (Table 1).

### Sand fly collection

Sand flies were collected using modified *Falcão* light traps [25], between 06:00 p.m. and 06:00 a.m., for 24 months (May/2015 to April/ 2017), totalizing 864 hours of sample effort for each area. Collections were performed simultaneously, at the three sample sites. In order to identify the species, the structures of the head, thorax and abdomen were used, with emphasis on the genitalia, following the classification proposed by Galati [26]. The abbreviation of the genera followed Marcondes [27].

### Data analysis

The evaluation of the most abundant species according to their spatial distribution was performed using the Standardized Index of Species Abundance (SISA), according to Roberts e Hsi [28], where values ≥1 correspond to the most abundant species. To analyze the diversity of species of each area, the Shannon Weaver Diversity Index (*H*) was used [29]. The measure of dominance/evenness of species found in the community was calculated using the Pielou’s Index (*J*). This index ranges from 0 to 1, where values close to 1 demonstrate uniformity at the site and reveal whether only one species is dominant or if species are in equilibrium [29].

Diversity profiles by the Rényi series provide different diversity measures to compare equivalently the collection areas. This index was calculated using the vegan package [30]. The diversity value is equal to the number of species in the sample when the parameter α = 0. For α = 1, the diversity value corresponds to the Shannon index; and if considered α = 2, to Simpson's index [31, 32, 33].

The degree of association between climatic variables (temperature and accumulated rainfall) and the number of sand fly specimens form each area (forest, rural and urban) were evaluated by the Spearman’s correlation. Due to the non-homogenous absolute frequency of species among the collection sites, correlation analyzes were conditioned to the factor "collection site".

The Wilcoxon test was used to verify statistical differences between sex of sand flies. Taxonomic richness and abundance of individuals were compared between the three collection areas using Kruskal-Wallis and, Wilcoxon as post-test. In all analyzes, the *p* value used to demonstrate statistical significance was 0.05. The analyzes were conducted in software R version 3.2.0 [34].

To compare the three sampled areas a similarity matrix was used, calculated by the Bray-Curtis similarity index demonstrated through a dendrogram. This dendrogram uses similarity matrix values, based on distances calculated from differences between sample densities for each species, using the BioDiversity Pro program [35].

To confirm sample sufficiency and species richness, the species accumulation curve was calculated using the EstimateS program, version 9.1.0 with 1,000 randomizations of the data set (sample-based) and 95% confidence interval [36]. To calculate the Synanthropic Index (SI) of Nuorteva [15], the following formula was used:

$$SI=\frac{\left(2a+b-2c\right)}{2}$$

where:

- ‘a’ represents the percentage of individuals of a given species collected in the urban area;
- “b” stands for the percentage of individuals of this same species collected in the rural area; and
- ‘c’ represents percentage of individuals of this species collected in the forest area.

The Synanthropic Index of Nuorteva (SI) ranges from +100 to -100. Negative values indicate aversion to the human environment while positive values represent a greater preference for anthropogenic environments [15]. This index was calculated for species that presented n> 10.

To generate georeferenced maps, shapefiles were obtained from Intituto Brasileiro de Geografia e Estatística website (http://www.ibge.gov.br) [37] and U.S. Geological Survey [38], and the species with their respective record coordinates were inserted using QGIS 2.18.10 software [39] and SPRING software [40].

To compare species richness among the three biomes, a Venn diagram was designed in the ClickCharts 3.01 software [41], highlighting the number of unique species and that of common species among the areas.

### Weather data

The climatic data referring to the study period were extracted from the database of the Center for Monitoring Weather, Climate and Water Resources of Mato Grosso do Sul, which is linked to the National Institute of Meteorology (INMET) [42].

### Ethical statement

The research group has a permanent license for the collection of zoological material issued by the Instituto Brasileiro de Meio Ambiente e dos Recursos Naturais (IBAMA SISBio 25952–1). Field studies were carried out on 3 private properties, the owners of which gave permission to conduct the study in their respective intra and peridomiciliary areas. In addition, the field studies did not involve any endangered or protected species.

## Results

Fourteen phlebotomine species in eight genera were collected during a 24-month period: *Brumptomyia brumpti*, *Evandromyia aldafalcaoae*, *Ev*. *corumbaensis*, *Ev*. *lenti*, *Ev*. *sallesi*, *Ev*. *walkeri*, *Lutzomyia cruzi*, *Lu*. *forattinii*, *Martinsmyia oliveirai*, *Micropygomyia peresi*, *Nyssomyia whitmani*, *Psathyromyia bigeniculata*, *Pa*. *hermanlenti* and *Sciopemyia sordellii* (Table 2). This is the first report of *Pa*. *hermanlenti* presence in Corumbá.

**Table 2:** Absolute and relative frequencies of sand fly species in forested (*n* = 250), rural (*n* = 3420), and urban (*n* = 3543) areas and all three areas combined (*n* = 7213). Shannon index (*H*), Pielou index (*J*), and standardized index of species abundance (SISA). Corumbá, Mato Grosso do Sul, Brazil. May 2015 to April 2017.

Species	Forested area		Rural area		Urban area		Total		SISA
	No	%	No	%	No	%	No	%	
*Br*. *brumpti*	–	–	64	1.87	1	0.03	65	0.90	0.47
*Ev*. *aldafalcaoae*	–	–	1	0.03	–	–	1	0.01	0.04
*Ev*. *corumbaensis*	38	15.20	94	2.75	12	0.34	144	2.00	0.84
*Ev*. *lenti*	1	0.40	1	0.03	–	–	2	0.03	0.25
*Ev*. *sallesi*	–	–	2	0.06	1	0.03	3	0.04	0.77
*Ev*. *walkeri*	1	0.40	–	–	–	–	1	0.01	0.01
*Lu*. *cruzi*	165	66.00	2309	67.52	3488	98.44	5962	82.66	1.00
*Lu*. *forattinii*	18	7.20	112	3.27	12	0.34	142	1.97	0.77
*Mi*. *peresi*	23	9.20	809	23.65	29	0.82	861	11.94	0.94
*Mt*. *oliveirai*	2	0.80	15	0.44	–	–	17	0.24	0.31
*Ny*. *whitmani*	–	–	1	0.03	–	–	1	0.01	0.01
*Pa*. *bigeniculata*	2	0.80	9	0.26	–	–	11	0.15	0.30
*Pa*. *hermanlenti*	–	–	1	0.03	–	–	1	0.01	0.04
*Sc*. *sordellii*	–	–	2	0.06	–	–	2	0.03	0.04
**Total**	250	100.00	3420	100.00	3543	100.00	7213	100.00	–
**Shannon index (*H*)**	1.0906		0.9487		0.0980		–		–
**Pielou index (*J*)**	0.5244		0.3698		0.0546		–		–

Br.: Brumptomyia; Ev.: Evandromyia; Lu.: Lutzomyia; Mi.: Micropygomyia; Mt.: Martinsmyia; Ny.: Nyssomyia; Pa.: Psathyromyia; Sc.: Sciopemyia.

**Fig 2:**
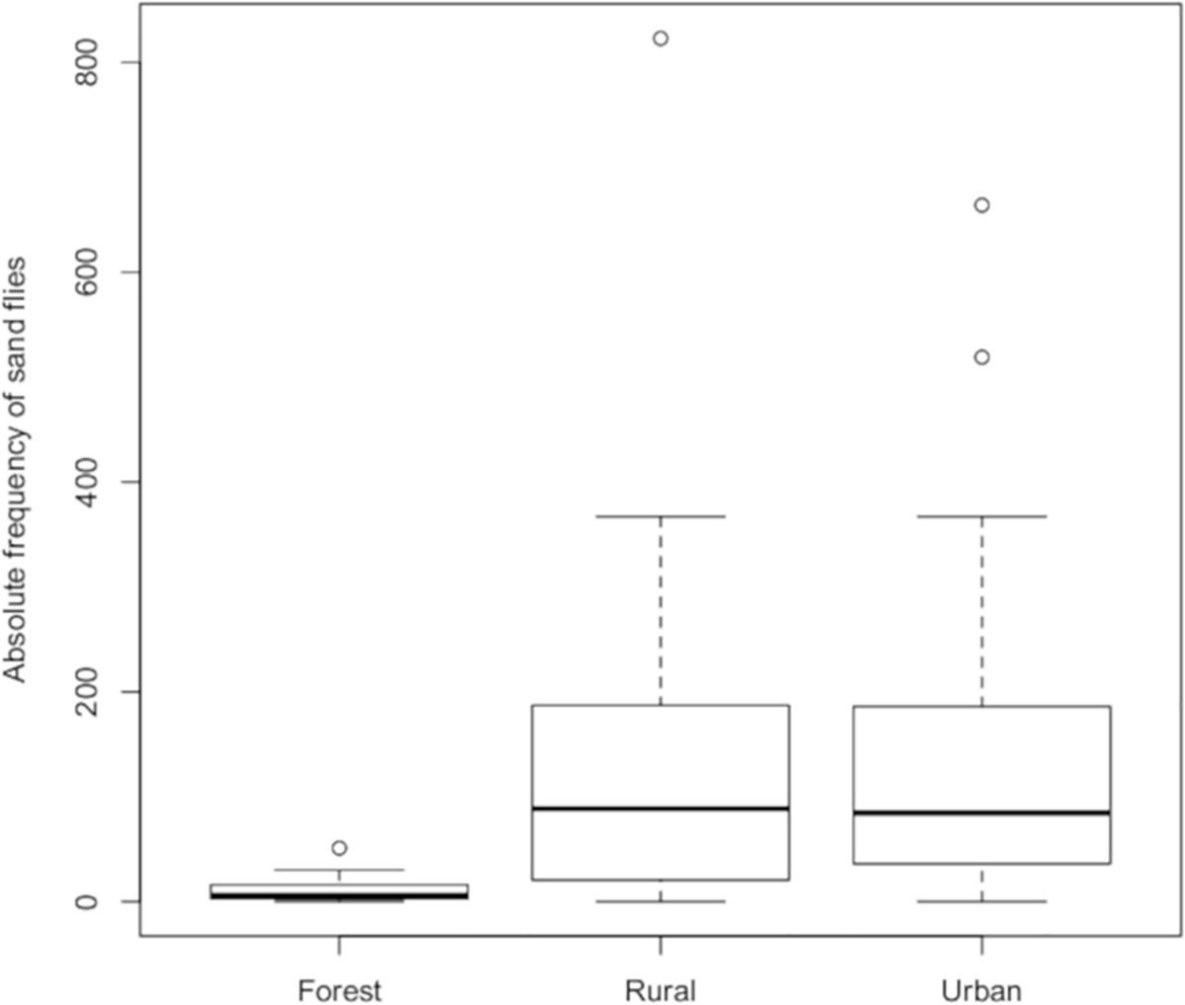
Absolute frequency of sandflies in three areas (forested, rural, urban). Corumbá, Mato Grosso do Sul, Brazil. May 2015 to April 2017.

Table 2 shows the absolute and relative frequencies of sand flies. Significant differences were found between the forested and the urban area (*W* = 81.5; *p* < 0.0001), as well as between the forested and the rural area (*W* = 71; *p* < 0.0001) (Fig 2).

**Fig 3:**
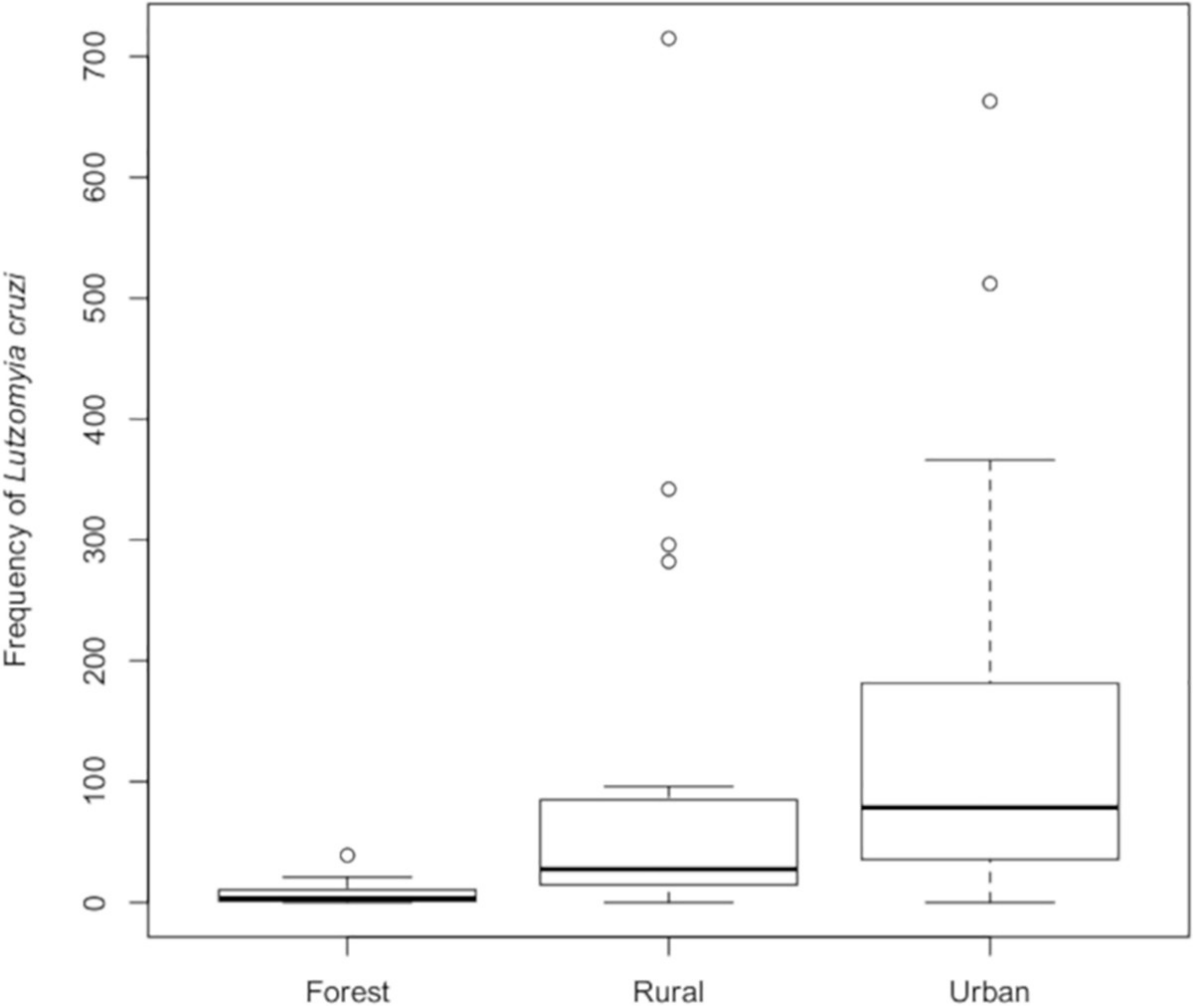
Absolute frequency of *Lutzomyia cruzi* in three areas (forested, rural, urban). Corumbá, Mato Grosso do Sul, Brazil. May 2015 to April 2017.

The species *Lu*. *cruzi* (standardized index of species abundance, SISA = 1.00) was the most abundant species, detected in all ten sampling plots and on all 24 months investigated, accounting for 82.66% of all sand flies captured. It was also the most frequent species in all three areas, representing over 50% of the number of sand flies collected from each environment (66.00%, 67.52%, and 98.44% for forested, rural, and urban areas, respectively) (Table 2). Significant differences were observed for *Lu*. *cruzi* between forested and rural areas (*W* = 85; *p* < 0.0001) and between forested and urban areas (*W* = 71.5; *p* < 0.0001) (Fig 3).

**Fig 4:**
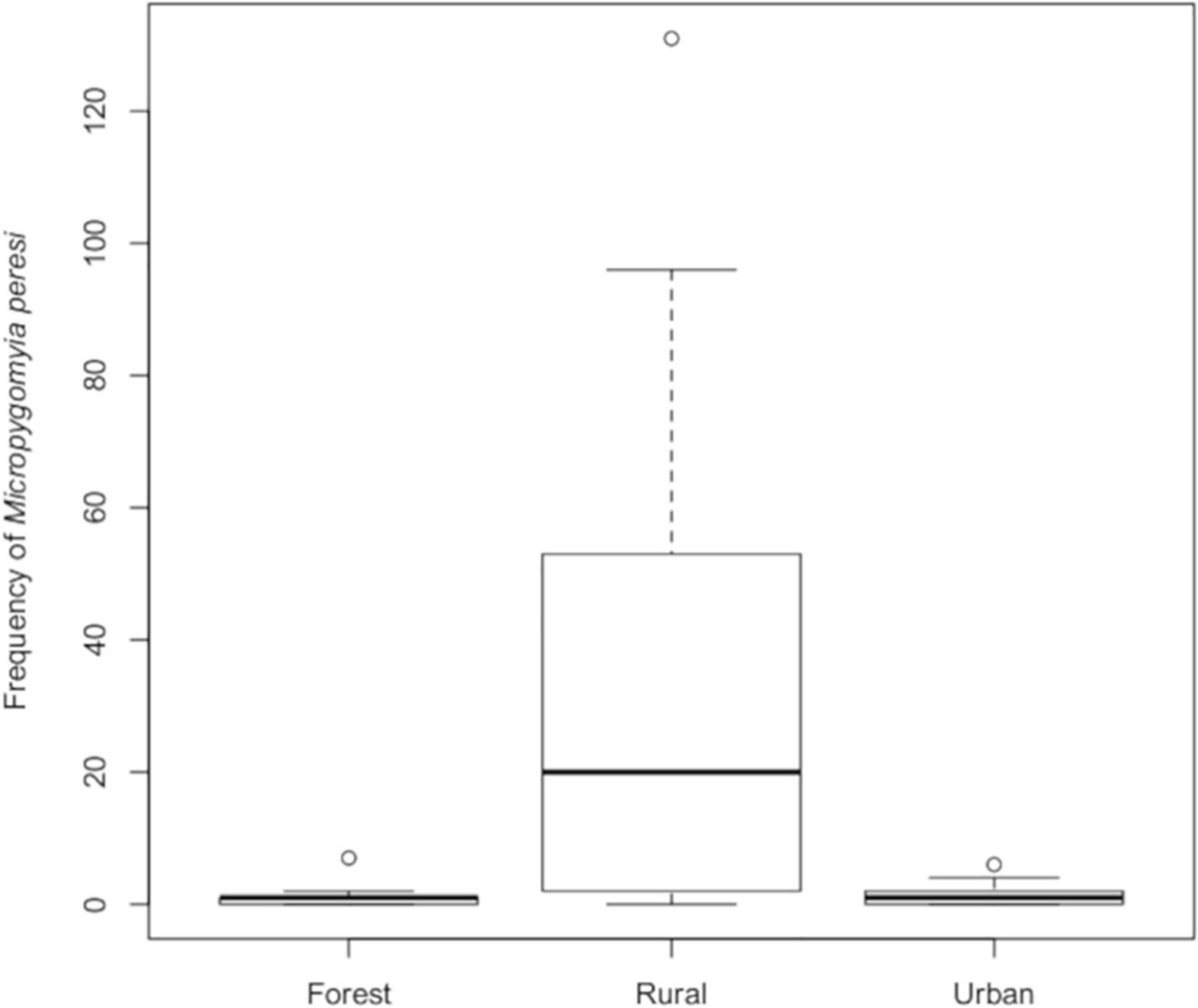
Absolute frequency of *Micropygomyia peresi* in three areas (forested, rural, urban). Corumbá, Mato Grosso do Sul, Brazil. May 2015 to April 2017.

*Micropygomyia peresi*, which also ranked second in species abundance (SISA = 0.94), was the second most frequent species (11.94% of the total), both in the rural (23.65%) and the urban area (0.82%) (Table 2 and Fig 4). No population homogeneity was observed for *Mi*. *peresi* between forested and rural areas (*W* = 71.5, *p* < 0.0001) or between rural and urban areas (*W* = 492, *p* < 0.0001) (Fig 4).

**Fig 5:**
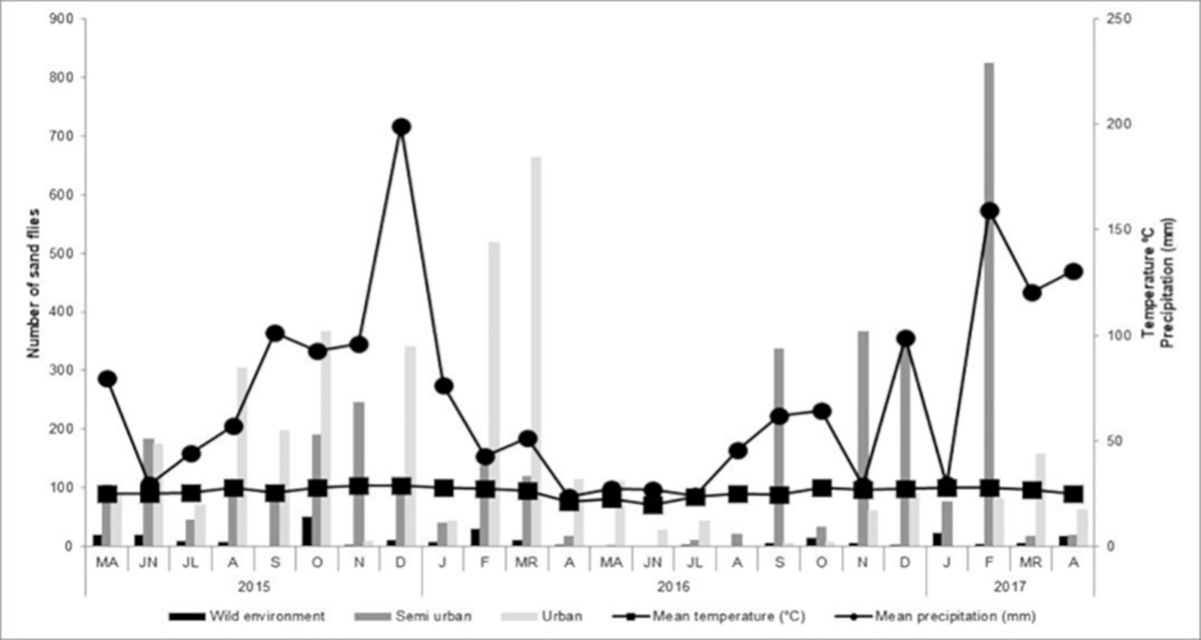
Temperature, precipitation, and seasonal distribution of sand flies in three areas (forested, rural, urban). Corumbá, Mato Grosso do Sul, Brazil. May 2015 to April 2017.

Fig 5 shows the distribution of sand flies during the 24-month sampling period and the relationships between distribution, precipitation, and temperature. Collection numbers peaked in the rainy, warm season (February-March, 2016, in the urban area and February, 2017, in the rural area). A sharp decrease in species density was observed in the dry, cold season. In forested and urban areas, the absolute frequency of sand flies did not correlate significantly with temperature or precipitation (forest temperature: *r* = 0.3841, *p* = 0.0638; forest precipitation: *r* = 0.03797, *p* = 0.8602; urban temperature: *r* = 0.1204, *p* = 0.5749; urban area precipitation: *r* = 0.2235; *p* = 0.2936). In the rural environment, absolute frequency correlated with temperature (*r* = 0.5086; *p* = 0.0121), but not with precipitation (*r* = 0.2782; *p* = 0.1874) (Fig 5).

**Fig 6:**
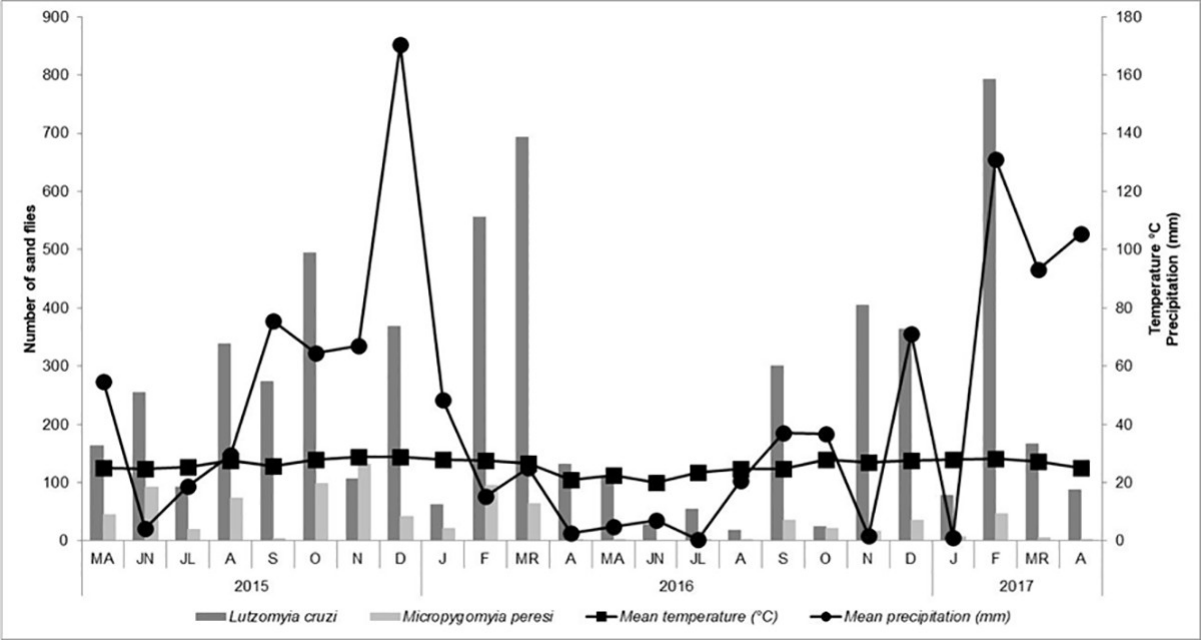
Temperature, precipitation, and seasonal distribution of *Lutzomyia cruzi* and *Micropygomyia peresi*, Corumbá, Mato Grosso do Sul, Brazil. May 2015 to April 2017.

With regard to seasonality of the two most abundant species, peaks for *Lu*. *cruzi* subsequent to the rainy season (February-March) were observed in both years (Fig 6). No significant correlations were observed between *Lu*. *cruzi* and temperature (*r* = 0.2641; *p* = 0.2123) or rainfall (*r* = 0.0425; *p* = 0.8435) in the forested area or between *Lu*. *cruzi* and temperature (*r* = 0.1208; *p* = 0.5736) or rainfall (*r* = 0.2270; *p* = 0.2861) in the urban area. In the rural area, the absolute frequency of *Lu*. *cruzi* correlated significantly with temperature (*r* = 0.4199; *p* = 0.0410), but not with rainfall (*r* = 0.2619; *p* = 0.2162). For *Mi*. *peresi*, a significant correlation with temperature (*r* = 0.5582; *p* = 0.0045) was detected in the rural area (Fig 6).

**Fig 7:**
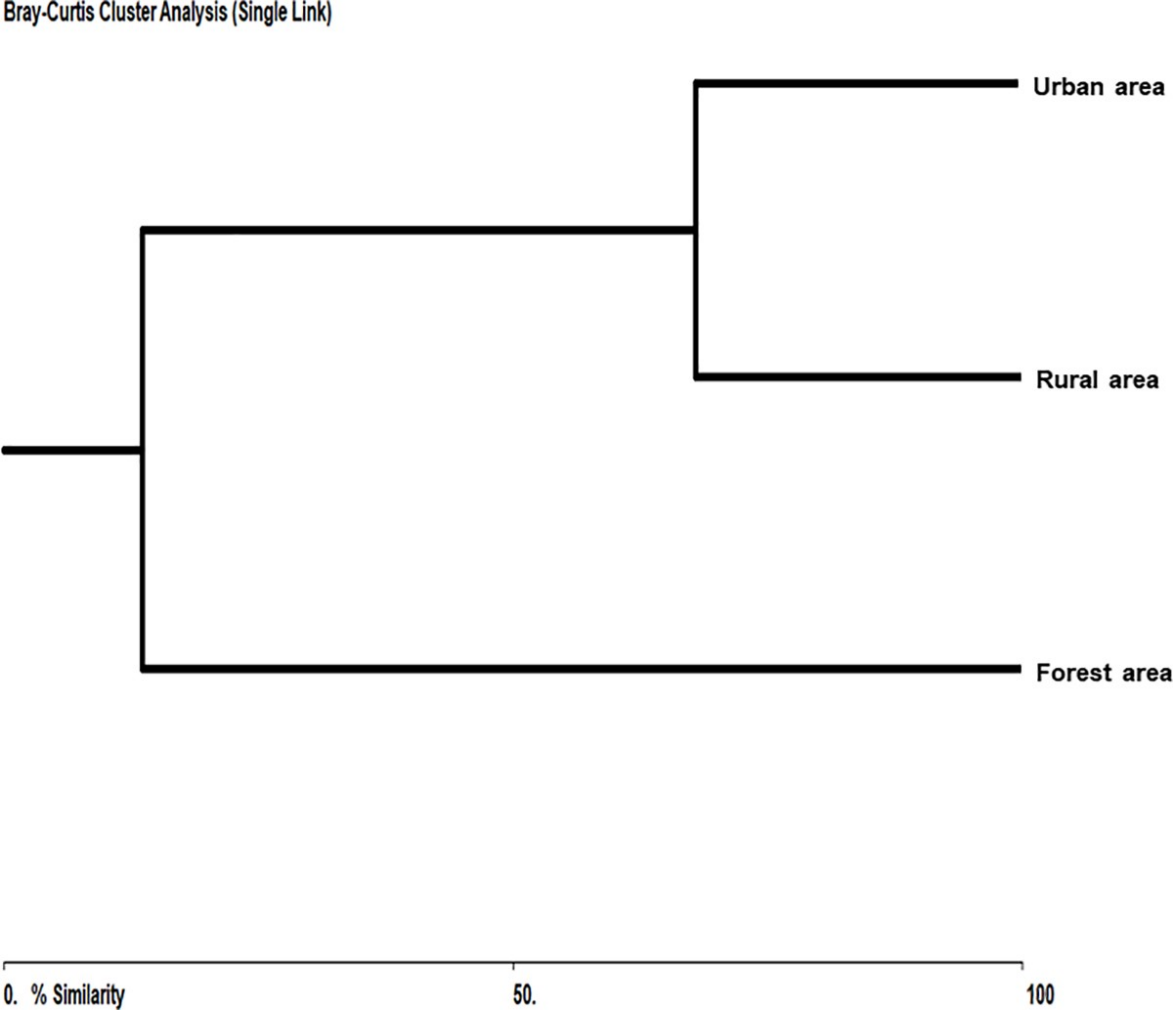
Similarities among three areas (forested, rural, urban). Corumbá, Mato Grosso do Sul, Brazil. May 2015 to April 2017.

No significant correlations were observed in the other areas. Sand fly frequency was highest in the rural environment (47.41%) and the urban area (49.11%), in contrast with the forested environment (3.46%). Diversity (*H* = 1.0906) and equitability (*J* = 0.5244) were highest in the forested area (Shannon diversity index) (Table 2). Similarity was highest between the rural and urban areas (67.90%) and lowest between the forested and urban areas (11.17%) (Fig 7).

**Fig 8:**
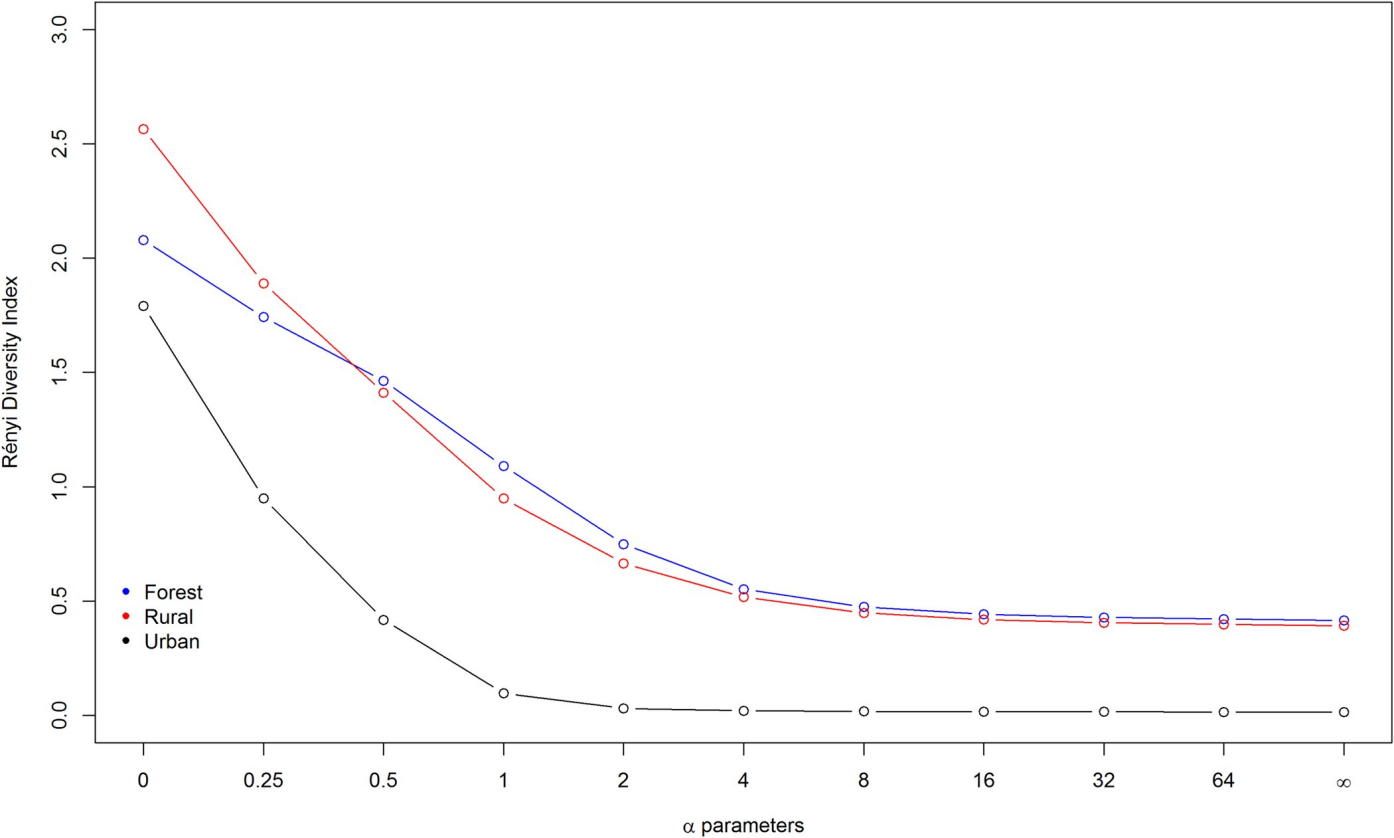
Species richness and diversity profiles of Rényi, showing Shannon index (*H*) (*α* = 1) and Simpson index (S) (*α* = 2) diversity in the three sampled areas (forest, rural and urban). Corumbá, Mato Grosso do Sul, Brazil. May 2015 to April 2017.

Although initially a larger species richness can be observed in the rural area (*n* = 13 species) in relation to the others (forest: *n* = 8 species, urban: *n* = 6 species) (Fig 8) and the profile of Rényi (*α* = 0) has been higher in the rural area, the rural and forest areas are non-comparable because the diversity profiles are intersecting. In any case, the urban area presented less diversity in comparison to the others.

**Fig 9:**
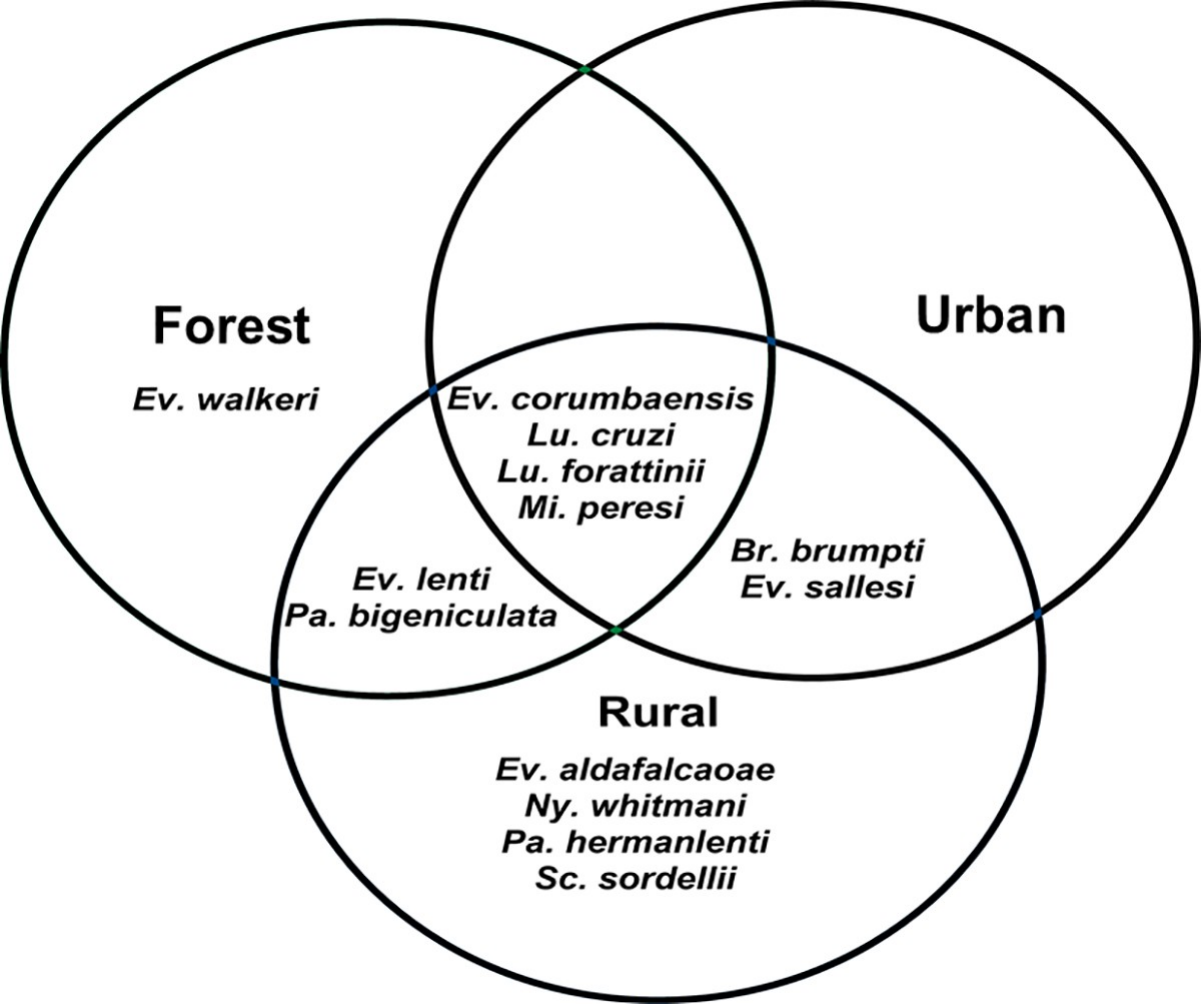
Distribution of sandfly species in three areas (forested, rural, urban). Corumbá, Mato Grosso do Sul, Brazil. May 2015 to April 2017.

*Evandromyia corumbaensis*, *Lu*. *cruzi*, *Lu*. *forattinii*, and *Mi*. *peresi* were recorded in all three areas. *Evandromyia walkeri* was found exclusively in the forested area and *Ev*. *aldafalcaoae*, *Ny*. *whitmani*, and *Sc*. *sordellii* were found in the rural area alone (Table 2 and Fig 9).

Among the 14 recorded species, synanthropy index were calculated for the seven species. Based on the synanthropy index, *Lu*. *cruzi* was found to have a strong preference (+75.09) for urban environments, while *Br*. *brumpti*, *Mi*. *peresi*, *Lu*. *forattinii*, and *Mt*. *oliveirai* were categorized as having a preference for these environments. Only *Ev*. *corumbaensis* was categorized as independent from urban environments (Table 3).

**Table 3:** Synanthropic index for male and female sand flies (*n* > 10) in three areas (forested, rural, urban). Corumbá, Mato Grosso do Sul, Brazil. May 2015 to April 2017.

Species	SI	The significance of the different degrees of synanthropic index	Values	
*Lu*. *cruzi*	+75.09	Strong preference for urban environments	+90	+65
*Br*. *brumpti*	+50.77	Preference for urban environments	+65	+20
*Mi*. *peresi*	+47.68	Preference for urban environments	+65	+20
*Lu*. *forattinii*	+35.20	Preference for urban environments	+65	+20
*Mt*. *oliveirai*	+32.36	Preference for urban environments	+65	+20
*Ev*. *corumbaensis*	+14.58	Independent from urban environments	+20	0

Br.: Brumptomyia; Ev.: Evandromyia; Lu.: Lutzomyia; Mi.: Micropygomyia; Mt.: Martinsmyi.

A total of 5997 males (83.11%) and 1216 females (16.89%) were collected. Males were more frequent in all three areas (male-to-female ratio: 4.93:1). Pooling all species together revealed a significant difference in absolute frequency between males and females (*W* = 490; *p* < 0.0001). For *Lu*. *cruzi*, the most abundant species in this study, an 8:1 male-to-female ratio (5299 males, 663 females; Table 4) was found, representing a significant difference (*W* = 519.5; *p* < 0.001). Males of *Lu*. *cruzi* represented 78.32%, 75.94%, and 99.12% of individuals for this species in the forested, rural, and urban areas, respectively (Table 5).

**Table 4:** Distribution of male and female specimens (*n* = 7213). Corumbá, Mato Grosso do Sul, Brazil. May 2015 to April 2017.

Species	Forested area				Rural area				Urban area				Total			
	M	%	F	%	M	%	F	%	M	%	F	%	M	%	F	%
*Br*. *brumpti*	–	–	–	–	37	0.51	27	0.37	–	–	1	0.01	37	0.51	28	0.40
*Ev*. *aldafalcaoae*	–	–	–	–	1	0.01	–	–	–	–	–	–	1	0.01	–	–
*Ev*. *corumbaensis*	6	0.08	32	0.44	21	0.29	73	1.01	2	0.03	10	0.14	29	0.40	115	1.60
*Ev*. *lenti*	–	–	1	0.01	–	–	1	0.01	–	–	–	–	–	–	2	0.03
*Ev*. *sallesi*	–	–	–	–	–	–	2	0.03	1	0.01	–	–	1	0.01	2	0.03
*Ev*. *walkeri*	1	0.01	–	–	–	–	–	–	–	–	–	–	1	0.01	–	–
*Lu*. *cruzi*	112	1.55	53	0.73	2017	28.00	292	4.05	3170	43.95	318	4.41	5299	73.46	663	9.20
*Lu*. *forattinii*	11	0.15	7	0.10	62	0.86	50	0.7	11	0.15	1	0.01	84	1.16	58	0.80
*Mi*. *peresi*	10	0.14	13	0.18	503	6.97	306	4.24	14	0.20	15	0.21	527	7.31	334	4.63
*Mt*. *oliveirai*	2	0.03	–	–	7	0.10	8	0.11	–	–	–	–	9	0.12	8	0.11
*Ny*. *whitmani*	–	–	–	–	1	0.01	–	–	–	–	–	–	1	0.01	–	–
*Pa*. *bigeniculata*	1	0.01	1	0.01	6	0.08	3	0.04	–	–	–	–	7	0.10	4	0.06
*Pa*. *hermanlenti*	–	–	–	–	1	0.01	–	–	–	–	–	–	1	0.01	–	–
*Sc*. *sordellii*	–	–	–	–	–	–	2	0.03	–	–	–	–	–	–	2	0.03
Total		1.97		1.47		36.84		10.6		44.34		4.78	5997	83.11	1216	16.89

Br.: Brumptomyia; Ev.: Evandromyia; Lu.: Lutzomyia; Mi.: Micropygomyia; Mt.: Martinsmyia; Ny.: Nyssomyia; Pa.: Psathyromyia; Sc.: Sciopemyia.

**Table 5:** Distribution of male and female specimens (*n* = 7213), by area (forested, rural, urban). Corumbá, Mato Grosso do Sul, Brazil. May 2015 to April 2017.

Species	Forested area				Rural area				Urban area			
	M	%	F	%	M	%	F	%	M	%	F	%
*Br*. *brumpti*	–	–	–	–	37	1.39	27	3.53	–	–	1	0.29
*Ev*. *aldafalcaoae*	–	–	–	–	1	0.04	–	–	–	–	–	–
*Ev*. *corumbaensis*	6	4.20	32	29.91	21	0.79	73	9.56	2	0.06	10	2.90
*Ev*. *lenti*	–	–	1	0.93	–	–	1	0.13	–	–	–	–
*Ev*. *sallesi*	–	–	–	–	–	–	2	0.26	1	0.03	–	–
*Ev*. *walkeri*	1	0.70	–	–	–	–	–	–	–	–	–	–
*Lu*. *cruzi*	112	78.32	53	49.54	2017	75.94	292	38.22	3170	99.12	318	92.17
*Lu*. *forattinii*	11	7.69	7	6.54	62	2.33	50	6.54	11	0.35	1	0.29
*Mi*. *peresi*	10	6.99	13	12.15	503	18.94	306	40.05	14	0.44	15	4.35
*Mt*. *oliveirai*	2	1.40	–	–	7	0.26	8	1.05	–	–	–	–
*Ny*. *whitmani*	–	–	–	–	1	0.04	–	–	–	–	–	–
*Pa*. *bigeniculata*	1	0.70	1	0.93	6	0.23	3	0.40	–	–	–	–
*Pa*. *hermanlenti*	–	–	–	–	1	0.04	–	–	–	–	–	–
*Sc*. *sordellii*	–	–	–	–	–	–	2	0.26	–	–	–	–
Total	143	100	107	100	2656	100	764	100	3198	100	345	100

Br.: Brumptomyia; Ev.: Evandromyia; Lu.: Lutzomyia; Mi.: Micropygomyia; Mt.: Martinsmyia; Ny.: Nyssomyia; Pa.: Psathyromyia; Sc.: Sciopemyia.

**Fig 10:**
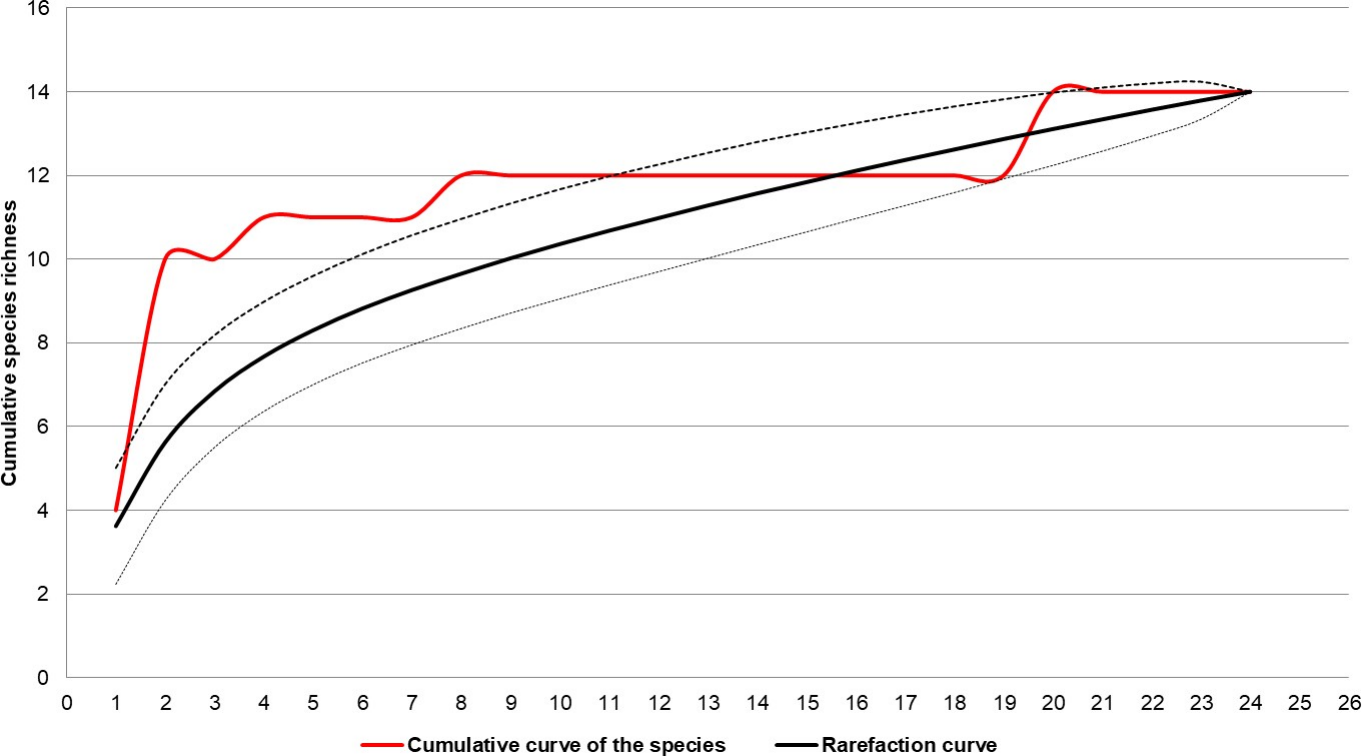
Cumulative curve of sandfly species in forested, rural, and urban areas (solid red line), 95% confidence intervals (dashed lines), and rarefaction curve (solid black line). Corumbá, Mato Grosso do Sul, Brazil. May 2015 to April 2017.

The results for sampling adequacy, based on the curve of species accumulation for the three areas, did not reveal apparent stabilization, thus failing to attain the predicted asymptote, suggesting the need for a greater sampling effort to record additional species (Fig 10).

## Discussion

This is the first study of sand fly synanthropy conducted in Midwest Brazil. The species *Lu*. *cruzi* had exhibited high synanthropic index and high population density. The Pantanal floodplain comprises both terrestrial and aquatic environments harboring a huge diversity of dipterous species, particularly species that, although restricted to wild environments, act as vectors of human pathogens. Destruction of the original vegetation, environmental imbalance, deforestation, agricultural development, human migration, and poor-quality, unplanned urbanization cause wild vectors and reservoirs of several parasites to adapt to rural and urban environments. The Pantanal floodplain is no exception to this phenomenon [6, 43].

Corumbá county has played a historical role in the study of leishmaniasis, as the place of diagnosis of the first human case of visceral leishmaniasis in Brazil, in 1913 [44]. In the 1980s, Nunes et al. [45] detected the prevalence of *Leishmania* sp. among dogs in the county, with cases of canine leishmaniasis recorded throughout the urban area. At the time, collection efforts revealed the phlebotomine fauna to comprise eight species, including *Lu*. *cruzi*, *Lu*. *forattinii*, and *Ev*. *corumbaensis* [46]. Successive faunal studies conducted in Corumbá showed an increase in the number of detected species over time, but a reduction in the number of individuals in some populations. In 2014, Casaril *et al*. [47] reported the presence of 12 species, four of which were new. Recently, Oliveira *et al*. [48] found 13 species.

Since the earliest studies of phlebotomines in the Pantanal region [46], *Lu*. *cruzi* had exhibited high anthropophilia and high population density. In the present study, this species was frequent in all plots throughout the collection period, accounting for 82.66% of the sand flies collected. *Lutzomyia cruzi*, the predominant vector species in Corumbá, has been reported as a vector for *L*. *infantum* [49–52]. Its synanthropic index indicates a strong preference for urban environments, demonstrating the adaptability of this species for eclectic feeding habits, which occurs in both preserved and disturbed environments.

In the present study, *Mi*. *peresi*, the second most prevalent species, was frequently found intradomestically. The species, often reported from environments with human presence in Mato Grosso do Sul [47, 53, 54] had a positive synanthropic index (+47.68) in the present study, indicating a preference for urban environments. Although the genus *Micropygomyia*—believed to feed on cold-blooded animals, such as lacertids [46]—has not been associated with *Leishmania* transmission to humans or animals, fauna monitoring should be periodically performed to detect the presence and circulation of *Leishmania* sp. in these sand flies.

In contrast with previous investigations conducted in the same county [46, 47, 55, 56], our study detected a sharp decrease in the density of *Lu*. *forattinii*, even in an urban environment containing chicken coops. Density of this species was highest in the rural area. Intense competition between species can cause one of the species to decline or even disappear [57]. It has been suggested that, owing to strong phylogenetic proximity [55], *Lu*. *cruzi* and *Lu*. *forattinii* share the same resources, implying competition. Despite a decline in density, *Lu*. *forattinii* remains a public health concern, not only because *Leishmania* DNA has been detected in females, but also owing to the high anthropophilia exhibited by this species [50]. The preference for urban environments (synanthropic index: +35.20) further exacerbates the concern over *Lu*. *forattinii* in the epidemiology of leishmaniasis.

*Evandromyia corumbaensis* and *Ev*. *sallesi*, two species belonging to the Cortelezzii complex, were collected in the present study. With type locality in Corumbá [58], *Ev*. *corumbaensis* exhibited eclectic behavior in the present study, having been recorded in forested and peridomestic areas (including chicken coops). Despite a low synanthropic index (+14.58: independence from anthropogenic environments), and although vector capacity has not been documented for *Ev*. *corumbaensis*, species belonging to the same complex, such as *Ev*. *sallesi*, have been reported as naturally infected with *L*. *infantum* promastigotes in Minas Gerais state [59], suggesting the importance of monitoring these species in the Pantanal floodplain. In the present study, *Evandromyia lenti* was found at a low density, comprising only females, recorded from better preserved environments. Natural infection with *L*. *braziliensis* [60] has been reported for *Evandromyia lenti*, a species often associated with domestic shelter in rural areas [61].

Of the areas investigated, the forest had the lowest density of sand flies (3.46%), corroborating findings by Teodoro *et al*. [62] (4.2%). In the present study, the forested areas had rocky soil with few litter layers, a feature that may have contributed to the low density of sand flies, since these insects depend on decaying organic matter for the development of immature forms. Despite having the lowest density, the forested area exhibited the highest diversity, as shown by the Shannon index (*H*), which combines not only richness and abundance of recorded species, but also balance between species [63] and these data were reinforced by Rényi diversity profile, these index is one of the most useful methods for ordering communities; it performs well irrespective of the species number of the community; the intersection of the diversity profiles is also well-indicated by this method [32]. *Lutzomyia cruzi* was the most frequent species in the forested area. *Evandromyia walkeri* was recorded only in this environment (one male specimen). Nieves *et al*. [64] associated *Ev*. *walkeri* with conserved areas in Venezuela, including specimens naturally infected with developmental forms of *Leishmania* sp., suggesting that this sand fly species may be involved in the transmission of tegumentary leishmaniasis agents. Casaril *et al*. [47] and Figueiredo *et al*. [65] reported a sustained presence of this species in peridomestic environments, particularly those with domestic animals.

The results obtained for the rural area (47.11% of total sampled sand flies) reveal the role played by intermediate environments in the maintenance of sand flies, since the features of rural environments tend to select species exhibiting an adaptive potential. Furthermore, rural environments provide conditions for the survival of sand flies (humidity, shade, decaying organic matter, domestic animals, and human dwellings near preserved forest). Vilela *et al*. [66] reported high diversity and high density in rural and urban environments, respectively, demonstrating the degree of adaptation that sand flies have been undergoing in response to anthropogenic changes, especially for *Lu*. *cruzi*.

Only male specimens were collected for *Ev*. *aldafalcaoae*, *Ny*. *whitmani* and *Pa*. *hermanlenti*, and only females for *Sc*. *sordellii*—all four species found exclusively in the rural area. *Evandromyia aldafalcaoae*, a species restricted to Mato Grosso do Sul, has been frequently collected from disturbed environments—peridomestically in the Pantanal sub-region of Nhecolândia [67] and in Corumbá county [47, 55], as well as indoors [65, 68]. *Nyssomyia whitmani*, a vector of etiological agents of cutaneous leishmaniasis in several Brazilian localities, has been frequently reported from Mato Grosso do Sul, especially where outbreaks of tegumentary leishmaniasis have occurred [61, 69, 70, 71].

*Sciopemyia sordellii* was found at a very low density in the present study. Casaril *et al*. [47] reported a decline of this species in Corumbá county, relative to a previous survey [46]. Urban expansion into forested areas can cause sand fly species to either disappear locally or successfully adapt to environmental changes, with an increase in relative abundance [57]. *Sciopemyia sordellii* is characteristic of rural areas and more preserved environments such as forests and caves [46, 47, 72]. In the present study, *Br*. *brumpti* occurred almost exclusively in the rural area, with a positive synanthropic index (+50.77: preference for urban environments). As yet, however, this genus does not pose epidemiological concern for leishmaniasis transmission.

*Psathyromyia bigeniculata*, a species with epidemiological importance, was found mainly in the rural area. The species has a wide geographic distribution and has been frequently reported from our study area [47, 48]. Reports of natural infection with flagellates [70, 73], as well as reports of vector competence in experimental transmission [74] and anthropophilic activity in various regions [61], have drawn attention to the importance of monitoring this species. *Psathyromyia hermanlenti* and *Mt*. *oliverai* were also found in the rural area, suggesting a preference for forested and rural environments [75–77].

The role of chicken coops in maintaining the biological cycle of *Leishmania* species in urban areas has been reported elsewhere [76,78–80]. In the present study, most *Lu*. *cruzi* specimens were collected from chicken coops, where decaying organic matter, humidity, and shelter facilitate breeding of this vector species, suggesting the potential of chicken coops to spread vector populations [81–83]. The three areas sampled proved heterogeneous for the presence or absence of sand fly species, and similarities were more pronounced for anthropogenically altered areas. Species composition can be influenced by geographic and meteorological conditions, but mostly by microclimate, increasing the likelihood of high similarity between anthropogenically altered habitats [84].

The species accumulation curve showed that the sampling effort was insufficient to reveal the richness of the sand fly, suggesting that richness can be increased in three sampled areas, mainly in the most preserved. *Lutzomyia cruzi* was collected on all months of sampling, both in the rainy and dry seasons, revealing the high plasticity of this species in the region. *Micropygomyia peresi* was absent from records only in June, 2016. *Lu*. *cruzi* populations peaked in October, February, and March, showing a trimodal pattern, as also observed by Galati *et al*. [46], Casaril *et al*. [47], and Fernandes *et al*. [71]. No periodicity was observed for rainfall, and sand fly frequency did not correlate significantly with this variable. Influence of temperature on *Lu*. *cruzi* and *Mi*. *peresi* densities was observed only for the rural area.

Males predominated significantly for most species, as shown by male-to-female ratios, corroborating results obtained by Casaril *et al*. [47]. The predominance of males may have resulted from the method of capture employed, since males are more strongly attracted to light traps [14]. Attracted by light before mating, males perform lekking, an aggregation behavior observed in *Lu*. *longipalpis* and *Lu*. *cruzi* [85,86]. Pheromones released to attract females also attract other male sand flies [87]. Courtship involves wing flapping in continuous circling movements (a behavior termed “love song”), attracting females receptive to copulation [88]. A number of male pheromone chemotypes have been identified, including 9-methyl-germacrene-B, 3-methyl-α-himachalene, and cembrene-1 [89–92]. Engorged females tend to shelter in poorly lit places; also, being heavier than males, these females are less likely to reach the height of light traps and be captured.

The variety of synanthropic behaviors of dipterans, such as flies and mosquitoes, has been reported. Few studies, however, have discussed this behavior for sand flies [93, 94]. Comparing three types of environment is useful to reveal the degree of adaptation of sand flies to anthropogenically altered areas and identify the selective pressures imposed by these changes [16]. The high synanthropic index obtained for *Lu*. *cruzi* indicates the overall high degree of adaptability of this species in occupying and breeding in areas subjected to distinct levels of change—a feature that raises concern, since controlling this species, which is a vector of *L*. *infantum*, a protozoan associated with high lethality in South America, is costly, burdensome, and often unsatisfactory. Elucidating the synanthropic behavior of sand flies yields information to assist in vector control by revealing the adaptive potential of these insects to areas with human presence and indicating the likelihood of disease emergence and endemism in these environments.
